# Energy for myelination: Implications for metabolic disturbances in multiple sclerosis pathology

**DOI:** 10.4103/NRR.NRR-D-24-01570

**Published:** 2025-05-06

**Authors:** Milton Guilherme Forestieri Fernandes, Jack P. Antel, Timothy E. Kennedy

**Affiliations:** Neuroimmunological Diseases and Glia Biology Research Group, Department of Neurology and Neurosurgery, Montreal Neurological Institute, McGill University, Montreal, QC, Canada

Myelin, made by oligodendrocytes (OLs) in the central nervous system (CNS), is essential for neural transmission. In particular, myelin facilitates communication across the long connections between different brain regions that form the white matter. Myelinated segments also provide metabolic intermediates to axons, supporting their demanding energetic needs. Genetic disorders that disrupt myelin formation result in progressive neurologic degeneration, referred to as leukodystrophies. Multiple sclerosis (MS) is considered an acquired disease, reflecting both genetic and environmental factors.

MS typically begins at a relatively young adult age. Symptoms vary depending on the area of the CNS affected and may include visual impairment, weakness, sensory loss, pain, and motor coordination deficits. Relapsing-remitting MS is the most common form, with relapses defined as intermittent episodes of neurologic disturbance lasting for at least 24 hours and new focal gadolinium enhancing lesions being the imaging counterpart. This disease course can evolve into a progressive phase referred to as secondary progressive MS. Relapses may or may not continue; the latter referred to as PIRA (progression in absence of relapse activity). Notably, the frequency of this progression appears to be decreasing in the current era of immunomodulatory suppressive therapy. In a minority of individuals, symptoms progress continuously from the outset without relapses, referred to as primary progressive MS (Compston and Coles, 2008).

Autoimmunity is central to MS pathology and the role of the immune system in causing OL injury has been extensively studied. These efforts have led to treatments that target the systemic immune aspects of MS and have been notably effective in limiting or preventing relapses. In contrast, addressing the progressive aspect of primary and secondary progressive MS remains a significant challenge. While autoimmune-related mechanisms in MS have been extensively investigated, a possible contribution of OL metabolic disruption to disease progression is emerging.

What initially causes MS remains unclear, but two main hypotheses have been proposed (outside-in and inside-out). The outside-in model suggests that a factor external to the CNS activates the peripheral immune system to then target OLs in the CNS, resulting in damage and disability. Evidence supporting the outside-in model includes that MS onset invariably requires previous exposure to Epstein-Barr virus, precedent that exposure to immunotherapy check-point inhibitors and tumor necrosis factor modulators can induce white matter inflammation, and that intense immune-ablation therapy prevents further relapses in MS but not disease progression in either MS or genetic white matter disorders. The inside-out model proposes that an internal factor within OLs initially disrupts the cells, triggering signals that then activate and engage the immune system. This inappropriate secondary autoimmune reaction damages OLs, leading to demyelination and MS-associated symptoms (Titus et al., 2020). A possibility to be considered is that an initial outside-in injury could trigger a secondary immune response, for example, epitope spreading as seen in the experimental allergic encephalomyelitis model (Miller and Eagar, 2001), thus combining the two pathogenic models to account for the relapsing disease course of MS.

Susceptibility to develop MS is influenced by both genetic and environmental factors. Numerous genes have been associated with MS, many of which are involved in immune function, such as polymorphisms in the major histocompatibility complex, which could influence autoimmunity (Compston and Coles, 2008). In contrast to immunity, genes associated with CNS resilience and neurocognitive reserve have been identified that influence MS severity and progression (Harroud et al., 2023).

Environmental risk factors for MS include vitamin D deficiency, which is key to immune system regulation and may also influence OLs directly; cigarette smoking, which may trigger T-cell activation in the lung; virus infections, particularly Epstein-Barr; and obesity, which is associated with chronic inflammation. MS is also approximately three times more prevalent in females than males (Rodríguez Murúa et al., 2022).

**Immune aspects of multiple sclerosis:** Immune-related mechanisms implicated in MS pathogenesis include contributions from both the adaptive and innate immune systems that involve the systemic and CNS compartments and their interactions. Environmental risk factors, such as viral infection and smoking, can activate antigen presenting cells in peripheral tissues. Activated antigen presenting cells then migrate to lymph nodes and activate lymphocytes, including CD4 and CD8 T cells. The activation of CD4 T cells is associated with genetic predispositions linked to the major histocompatibility complex. Once activated, CD4 T cells differentiate into Th1, Th2, or Th17 subsets, which produce pro-inflammatory cytokines and activate B cells and myeloid cells, causing harm to OLs. Activated B cells differentiate into plasma cells that secrete autoimmune antibodies. CD8 T cells, often detected in MS lesions, contribute to CNS damage by releasing granzymes, perforins, and Fas ligand, resulting in OL death. Myeloid cells, either systemic derived macrophages or their CNS counterparts, microglia, can modulate their phenotypic profile, adopting either pro-inflammatory or immunoregulatory roles. Additional immune cells implicated in MS pathology include γδ T cells, mast cells, and natural killer cells. While not canonically part of the immune system, astrocytes may contribute to MS by secreting cytokines and neurotoxic metabolites that promote inflammation and neurodegeneration. In addition, OL lineage cells under inflammatory and stress conditions can acquire antigen presenting capacity (Rodríguez Murúa et al., 2022).

An essential early step in neuroinflammation and MS autoimmunity is crossing the blood-brain barrier. In MS, CNS endothelial cells upregulate adhesion molecules that promote T cell infiltration. Mast cells may contribute to blood-brain barrier permeability by releasing histamine, tryptase, and activated matrix metalloproteinases, which can also be secreted by Th17 cells (Rodríguez Murúa et al., 2022).

Recovery after an MS relapse typically involves mechanisms that halt the neuroinflammatory immune reaction, followed by remyelination. Cessation of the immune reaction is mediated by inflammatory cytokines produced in the CNS, apoptosis of inflammatory cells, debris clearance by myeloid cells, and activation of regulatory T cells. Remyelination involves oligodendrocyte precursor cells that are activated by immunoregulatory factors, differentiate into OLs, and migrate to areas requiring myelination (Antel et al., 2019). Mature OLs engaged in remyelination have also been observed in MS lesions and the capacity for myelin regeneration has been demonstrated *in vitro* (Cui et al., 2017).

**Metabolic aspects of multiple sclerosis:** In addition to immune-mediated aspects, a well-characterized pathological feature of MS is decreased cerebral blood perfusion. Hypoperfusion can induce metabolic stress in the brain parenchyma, which may be particularly harmful to OLs. Imaging studies have demonstrated reduced cerebral blood perfusion in patients exhibiting initial MS symptoms (clinically isolated syndrome), relapsing-remitting MS, and progressive MS, with trends toward greater hypoperfusion in progressive cases. Imaging studies have identified hypoperfusion and hypoxia-like environments in MS lesions, particularly in progressive cases (Fernandes et al., 2024).

Potential mechanisms underlying hypoperfusion in MS involve dysregulation of the neurovascular unit, which can lead to ischemic-like conditions in the CNS parenchyma and harm OLs. Astrocytes provide crucial regulation, secreting endothelin-1, a potent vasoconstrictor, with elevated levels detected in MS patients and MS plaques, potentially causing hypoperfusion and OL metabolic stress. Additionally, astrocytes regulate vasodilation by modulating the concentration of extracellular K^+^. Axonal degeneration followed by mitochondria dysfunction and oxidative stress can also contribute to hypoperfusion in MS. Ischemic-like conditions may also be induced by excitotoxity caused by excessive glutamate release, which is observed in MS lesions (Fernandes et al., 2024).

**Interactions between metabolic and immune aspects:** Metabolic impairment and the reactive immune response are interconnected processes that can amplify the pathological environment in MS (**Figure 1**). Mitochondrial dysfunction in OLs can be triggered by inflammation and cytokines, such as interferon γ and tumor necrosis factor α. From a metabolic perspective, mitochondrial dysfunction in OLs can result in the release of cytokines that, in turn, activate microglia. Microcirculation dysfunction and inflammation of vessel walls further contribute to blood-brain barrier disruption, exacerbating hypoxic conditions in MS lesions (Fernandes et al., 2024). Further, the interplay between nutrient deprivation and pro-inflammatory cytokines can intensify OL stress, compounding the damage (Pernin et al., 2024).

**Figure 1:**
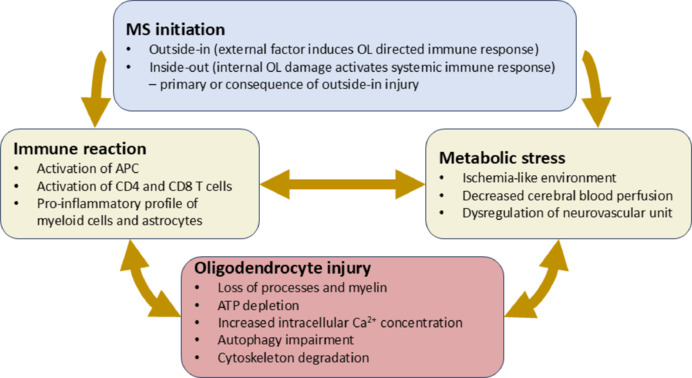
Metabolic components and interplay with the immune response in MS pathology. MS initiation may be triggered by factors external to the CNS (outside-in) or by factors in oligodendrocytes that activate an immune response (inside-out). Activation of APC, CD4 and CD8 T cells, and the conversion of myeloid cells and astrocytes to a pro-inflammatory profile can harm oligodendrocytes. Decreased cerebral blood perfusion and ischemic-like environments within brain parenchyma may be present in MS, possibly caused by dysregulation of cerebrovascular modulators. The immune response can aggravate metabolic stress, which in turn can exacerbate the immune reaction. These conditions lead to oligodendrocyte injury, with retraction of oligodendrocyte processes and myelin loss, ATP depletion, increased intracellular Ca^2+^ concentration, impaired autophagy, and cytoskeletal degradation, ultimately resulting in the loss of metabolic viability and cell death. APC: Antigen presenting cell; ATP: adenosine triphosphate; Ca: calcium; CD4 T: cluster of differentiation 4 thymocyte; CD8 T: cluster of differentiation 8 thymocyte; CNS: central nervous system; MS: multiple sclerosis; OL: oligodendrocytes.

**Metabolic stress in oligodendrocytes:** OLs are large highly specialized post-mitotic cells with particularly unique energy demands. OL metabolism is biased toward glycolysis. Rather than continuing to process glycolytic products for ATP production, they are shunted away from mitochondrial oxidative phosphorylation, to instead be used to build the macromolecules that support the lipid-rich structure of myelin, in addition to shipping lactate and pyruvate to axons to support their intense activity (Fernandes et al., 2024).

Cerebral hypoperfusion and ischemic-like conditions in brain parenchyma can impose significant metabolic stress on OLs, impairing their ability to perform essential functions due to insufficient energy availability. OLs require substantial energy to produce and maintain myelin and to support the high energetic demands of neurons. When deprived of nutrients, OLs and the myelin they support are compromised, leading to myelin loss. However, these processes have the potential to recover if adequate energy supplies are restored (Cui et al., 2017).

Human OLs exhibit considerable metabolic resilience, capable of surviving for days under extreme nutritional deprivation. They resist the activation of regulated cell death pathways, including apoptosis, ferroptosis, and mitochondrial permeability transition pore-driven necrosis. Exposure to pro-inflammatory cytokines such as tumor necrosis factor α and interferon γ, or glutamate-mediated excitotoxicity, does not induce human OL cell death *in vitro*. However, these conditions can lead to process retraction, compromising OL function (Pernin et al., 2022).

Human mature OLs are more resistant to cell death and are less metabolically active than human oligodendrocyte precursor cells and rodent OLs. This lower metabolic activity may contribute to their resistance to cell death. Additionally, human mature OLs exhibit a relatively higher expression of anti-apoptotic proteins compared to pro-apoptotic molecules, in contrast to human oligodendrocyte precursor cells and rodent OLs (Fernandes et al., 2021). Death of human mature OLs induced by metabolic stress is likely driven by ATP depletion. This increases intracellular Ca^2+^ to activate Ca^2+^-dependent proteases that degrade cytoskeletal components. ATP depletion also impairs autophagy, preventing the recycling of cellular components for energy production and ultimately results in the collapse of cellular viability. Further, myelin debris resulting from metabolic stress increases astrocytic and microglial phagocytotic activity and impacts immune interactions (Fernandes et al., 2023).

**Clinical and research perspectives:** Metabolic stress and cerebral hypoperfusion are potentially harmful conditions for OLs that are integral to the etiology of MS and should be considered when investigating mechanisms that underlie MS progression. Further studies are required to fully understand the functional relationships between cerebral hypoperfusion, inflammation, metabolism, and OL damage. In this context, treatments that target metabolic processes, such as metformin and biotin (Cui et al., 2020; Mohammadnia et al., 2024), should be carefully evaluated. Biotin, a cofactor of carboxylases involved in cellular respiration, may facilitate the capacity of OLs to cope with metabolic stress (Cui et al., 2020). Metformin treatment activates adenosine 5‘-monophosphate-activated protein kinase (AMPK), which can promote myelination; however, excessive AMPK activation can inhibit mTOR, thereby inhibiting protein synthesis and anabolic metabolism. Moreover, based on studies of OLs derived from different aged individuals, the sensitivity of AMPK to metformin changes with age, an aspect that needs to be taken into account in clinical settings (Mohammadnia et al., 2024). Promising opportunities exist to develop therapies to improve cerebral perfusion or mitigate the detrimental interplay between inflammation, hypoperfusion, and ischemic-like conditions. These strategies may also address age-related comorbidities, including hypertension, atherosclerosis, and small vessel disease. Changes in cerebral perfusion could serve as biomarkers for disease progression and treatment efficacy. Overall, adopting an etiological framework that incorporates metabolic impairments could enhance the understanding and management of MS.

*We acknowledge operating grant support held by JPA, Collaborative Network Award BRAVEinMS, Grant/Award Number: PA-1604-08492 (MG), and from the Multiple Sclerosis Society of Canada, Grant/Award Number: 1038154 (to TEK).*

## References

[R1] Antel JP, Lin YH, Cui QL, Pernin F, Kennedy TE, Ludwin SK, Healy LM (2019). Immunology of oligodendrocyte precursor cells in vivo and in vitro. J Neuroimmunol.

[R2] Compston A, Coles A (2008). Multiple sclerosis. Lancet.

[R3] Cui QL, Khan D, Rone M, T S Rao V, Johnson RM, Lin YH, Bilodeau PA, Hall JA, Rodriguez M, Kennedy TE, Ludwin SK, Antel JP (2017). Sublethal oligodendrocyte injury: a reversible condition in multiple sclerosis?. Ann Neurol.

[R4] Cui QL, Lin YH, Xu YKT, Fernandes MGF, Rao VTS, Kennedy TE, Antel J (2020). Effects of biotin on survival, ensheathment, and ATP production by oligodendrocyte lineage cells in vitro. PLoS One.

[R5] Fernandes MGF, Luo JXX, Cui QL, Perlman K, Pernin F, Yaqubi M, Hall JA, Dudley R, Srour M, Couturier CP, Petrecca K, Larochelle C, Healy LM, Stratton JA, Kennedy TE, Antel JP (2021). Age-related injury responses of human oligodendrocytes to metabolic insults: link to BCL-2 and autophagy pathways. Commun Biol.

[R6] Fernandes MGF, Mohammadnia A, Pernin F, Schmitz-Gielsdorf LE, Hodgins C, Cui QL, Yaqubi M, Blain M, Hall J, Dudley R, Srour M, Zandee SEJ, Klement W, Prat A, Stratton JA, Rodriguez M, Kuhlmann T, Moore W, Kennedy TE, Antel JP (2023). Mechanisms of metabolic stress induced cell death of human oligodendrocytes: relevance for progressive multiple sclerosis. Acta Neuropathol Commun.

[R7] Fernandes MGF, Pernin F, Antel JP, Kennedy TE (2024). From BBB to PPP: bioenergetic requirements and challenges for oligodendrocytes in health and disease. J Neurochem.

[R8] Harroud A (2023). Locus for severity implicates CNS resilience in progression of multiple sclerosis. Nature.

[R9] Miller SD, Eagar TN (2001). Functional role of epitope spreading in the chronic pathogenesis of autoimmune and virus-induced demyelinating diseases. Adv Exp Med Biol.

[R10] Mohammadnia A, Cui QL, Weng C, Yaqubi M, Fernandes MGF, Hall JA, Dudley R, Srour M, Kennedy TE, Stratton JA, Antel JP (2024). Age-dependent effects of metformin on human oligodendrocyte lineage cell ensheathment capacity. Brain Commun.

[R11] Pernin F, Luo JXX, Cui QL, Blain M, Fernandes MGF, Yaqubi M, Srour M, Hall J, Dudley R, Jamann H, Larochelle C, Zandee SEJ, Prat A, Stratton JA, Kennedy TE, Antel JP (2022). Diverse injury responses of human oligodendrocyte to mediators implicated in multiple sclerosis. Brain.

[R12] Pernin F, Cui QL, Mohammadnia A, Fernandes MGF, Hall JA, Srour M, Dudley RWR, Zandee SEJ, Klement W, Prat A, Salapa HE, Levin MC, Moore GRW, Kennedy TE, Vande Velde C, Antel JP (2024). Regulation of stress granule formation in human oligodendrocytes. Nat Commun.

[R13] Rodríguez Murúa S, Farez MF, Quintana FJ (2022). The immune response in multiple sclerosis. Annu Rev Pathol.

[R14] Titus HE, Chen Y, Podojil JR, Robinson AP, Balabanov R, Popko B, Miller SD (2020). Pre-clinical and clinical implications of “inside-out” vs. “outside-in” paradigms in multiple sclerosis etiopathogenesis. Front Cell Neurosci.

